# Impact of the Use of Media Devices within the Family Context on the Language of Preteens

**DOI:** 10.3390/children7120281

**Published:** 2020-12-09

**Authors:** Lucía Acebedo, Lucía Buil-Legaz, Daniel Adrover-Roig, Eva Aguilar-Mediavilla

**Affiliations:** 1 Faculty of Education, University of Balearic Islands, 07122 Palma, Spain; lucia.acebedo@ceipsaindioteria.cat; 2 Applied Pedagogy and Educational Psychology, Institute of Research and Innovation in Education (IRIE), University of Balearic Islands, 07122 Palma, Spain; lucia.buil@uib.es (L.B.-L.); daniel.adrover@uib.es (D.A.-R.)

**Keywords:** video games, input, education, adolescent, media, language acquisition, preteens

## Abstract

Several studies have found a negative impact of media use on the language of children under 5 years. This impact seems to be related to the linguistic input of their parents. However, less is known about the influence of media on language in preteens. This study aims to analyze the relationship between the use of media, the quantity of parental language input, and the linguistic level of preteens. We assessed the language level of 60 bilingual Spanish–Catalan preteens aged 11–12 years with four subtests of the Spanish version of the standardized clinical evaluation of language fundaments (CELF-5-Spanish) as well as media use at home through a multiple-choice questionnaire. Results showed lower language scores in preteens who had access to more media devices, who used them more frequently, and who talked less with their parents. Language scores were also significantly lower in preteens who used media devices to communicate compared to those who used it as a school aid or to learn new things. These results are not influenced by socioeconomic level, sex, chronological age, or family language. The present results highlight the negative impact of media use on the language level of older children, which is also related to the amount of linguistic input received from their parents.

## 1. Introduction

Nowadays, children and adolescents use media and technology in almost every area of their lives, such as social life and education contexts, being thus in contact with technological devices since very early in their development. We live in a society in which media is almost omnipresent and, in the case of children, it is also part of their learning process, which makes this topic of high scientific interest. In this vein, Prensky [1] labels “digital natives” those children and adolescents who are born and grow up with media devices fully available, which they use with a distinctive naturalness in comparison to other generations.

This situation happens to an extent in which even the youngest children and adolescents are exposed to media within their family context. Recent studies show that many parents use media for entertaining their children, which leads to long exposure periods to TV, tablets, and multiple technological devices [2]. Accordingly, seven out of ten Spanish children (71%) are exposed to media while eating—watching TV, or even using a tactile screen or a smartphone—as reported in the 5th CinfaSalud Study [3], which has been certified by the *Sociedad Española de Pediatría Extrahospitalaria y Atención Primaria* (SEPEAP; Spanish Society of outpatient paediatrics and primary attention).

At older ages, the use of devices increases, especially for watching videos and playing video games, since up to 98% of children between 10 and 14 years of age have a smartphone with an internet connection [4]. As suggested by the results presented by the Spanish National Institute of Statistics, the use of these devices increases rapidly from 26.1% of use at 10 years of age to 98% at 14 years of age [5,6].

As shown, several authors recognize that the use of technological devices is progressively becoming more common in our daily life. Even in the educational setting, the Spanish *Real Decreto* (Royal Ordinance) by which the basic curriculum in Primary Education is established [7] highlights the importance of CIT (Communication and Information Technology) at this educative stage, affirming that one of the seven basic competencies that a student must acquire is digital competence.

While the use of technological devices increases, language development and literacy seems to be affected. In this sense, some authors have suggested that the use of media can act as a strong predictor of cognitive delay [8]. For young adults, recent studies [9] have pointed out that current university students present a weakened expressive language, exhibiting errors that are more typical of earlier educative stages, mostly, grammatical mistakes. Moreover, some studies emphasize two environmental factors that can cause language delays: a diminished verbal parent–child interaction and children exposure to technological media (TV, videos, computers), both having clear implications for language development [10]. However, results are inconsistent, since there are studies that found that media exposure could also have positive effects on language development. In this sense, several authors report that viewing so-called educational programs at an early age of language acquisition could contribute to increasing vocabulary [11,12], although the acquisition of vocabulary seems to occur when the use of technologies is accompanied by the interaction with parents or caregivers [10]. Studies have even shown that using media for reading-related activities can be beneficial for learning [13] and that in certain cases, the use of certain applications can improve working memory [14]. Recent studies recognize that there is an excessive use of media and point to different adverse effects that this phenomenon can have on children and adolescents who abuse it [4,15]. As Christakis [16] pointed out, exposure to TV, and other audio-visual devices, is one of the main risk factors associated with language development in children under 5 years of age. In this sense, studies point out that the time spent with technological devices could be detrimental to the children’s language skills because it may displace language-enhancing activities and interaction with parents, resulting in less exposure to language input [17]. Previous studies describe a clear relation between watching TV from early ages (under 24 months of age) and language development problems [18]. For instance, as seen in a sample of 45 children, ranging from 18 months to 5 years of age, watching television from early ages, specifically before 2 years of age for more than 2 h per day, is considered as a risk factor for primary language development delays [2]. Moreover, this same study described an increment in the previous years of the number of requests for diagnostic evaluations regarding language maturation delays. In the same line, the American Academy of Pediatrics (APP) [19] found that children who start watching TV before they turn one year of age for more than 2 h per day are six times more likely to develop language problems. Likewise, Zimmerman, Christakis, and Meltzoff [12] demonstrated in a sample of children under 17 months of age that each daily hour watching television implied, on average, a diminution of 17 points in the *MacArthur-Bates Communicative Development Inventories* [20,21]. Another interesting longitudinal study, initiated in 2010 with more than 250 families, assessed the influence of screen exposure on developmental trajectories from childhood to adolescence. Their results showed that children already exposed to screens when they were 6 months old exhibited a lower cognitive and language development at 14 months of age, only 8 months later. Furthermore, no differences were found in terms of the content, educative or non-educative, to which they had been exposed [22].

Furthermore, the interaction quality, both verbal and non-verbal, between children and their parents is diminished by the simple presence of a nearby switched-on TV [23], suggesting an impact of screen presence on children even when they are not paying close attention to it. For this reason, several authors such as Pempek, Kirkorian, and Anderson [24] pointed out that television in the background can affect the quantity and quality of parent’s infant-direct speech, this being closely linked to children’s language development. These authors hypothesize that this negative influence can be generalized to all information and entertainment technological devices used by parents in the presence of their children. Hence, it is not only children’s exposition to screens and digital devices that is harmful to their development but also their parents’ exposition when they are present.

Therefore, the studies cited above suggest that media can affect language development, which is an interaction that seems mediated by the diminution of both the quantity and quality of the linguistic input provided by parents when media devices are present. Language stimulation is important since birth, and the quality of the linguistic input from caregivers, who are the first and primary interlocutors, is crucial for its adequate acquisition. As a child grows up, the relation between her or his language input and linguistic productions becomes more obvious, for the language that he or she learns depends on the input received from the environment over the years [25,26]. There is plenty of evidence regarding the importance of language input on children’s overall development, particularly in terms of language [27,28,29], and it seems that the use of media can act as a risk factor for this input when it is either reduced or impoverished. Nevertheless, some authors indicate that moderate amounts of media exposure may not be a negative influence on children’s language development. In this sense, it seems that co-viewing could act as a buffer regarding the relationship between media use and early language skills [30].

To summarize, the presence of technological devices within the family and educative settings can negatively affect language at early ages, because it can diminish the quantity and quality of linguistic input received by the child. However, studies assessing this topic in older children and adolescents are still needed.

Given the exponential growth of the use of media within the family and educative settings during adolescence, the importance of both parent’s language quantity and their implication on development [31,32,33], and the presence of studies suggesting that young adults’ language level is worsening, we sought to analyze the relationship between the use of media, the quantity of parental language input, and preteens’ language development. We aimed to explore the potential relationship between media use and the linguistic level of preteens of 11 and 12 years of age. Furthermore, we evaluated the association between the quantity of parental language input, as reported by preteens, and their actual language level.

## 2. Materials and Methods

### 2.1. Participants

The total sample included 60 preteens from different schools in Mallorca (Spain). All the participants were in the 5th or 6th grade of Primary Education (age in years: *M* = 11.63, *SD* = 0.486). Twenty-two of them (3 females) were 11 years old, and 38 (14 females) were 12 years old. All preteens were Catalan–Spanish bilinguals and used Catalan and Spanish as the school language. The language used in their family context was mostly Spanish except for seven participants who used mainly Catalan with their families. Regarding the socioeconomic status of the families, and considering the Spanish socioeconomic context, 33 participants fell into the middle category, and the other 27 were in the low category [34]. Hence, it could be considered that all families could afford to acquire technological devices and could use them daily if they wanted to.

None of the participants in our sample had a diagnosis of learning difficulties nor presented educative education needs. A summary of the sociodemographic data of the sample can be consulted in Table 1.

This study is part of a project funded by FEDER/Ministerio de Ciencia, Innovación y Universidades_Agencia Estatal de Investigación/EDU2017-85909-P, and it was approved by the Ethics Committee of the University of Balearic Islands (12 September 2017). All parents of the participants included in the sample provided written informed consent at the beginning of the study, all preteens consented as well to participate, and pertinent measures have been followed to maintain their anonymity.

### 2.2. Study Design

The present study followed a transversal design, and participants were evaluated with two tests that gathered information on the frequency and types of media use, language interaction with their parents, and participants’ expressive and receptive linguistic level.

### 2.3. Materials

A standardized validated questionnaire was used to obtain data regarding participant’s language level, and an ad hoc questionnaire served to quantify the frequency and type of media use within the family setting and the interaction frequency with their parents.

#### 2.3.1. CELF-5

To assess language level, four subtests from the Spanish version of the standardized clinical evaluation of language fundaments (CELF-5) were administered [35].

The CELF-5 is a clinical instrument designed for individual application that is composed of different subtests that allow assessing multiple linguistic aptitudes. Specifically, it is formed of 12 subtests and includes several complementary resources. For the present study, we used the four subtests that compound the Core Language Score (CLS) of the original Spanish version designed for participants between 9 and 15 years of age, which were:

- Word classes: This subtest evaluates the participant’s ability to understand relationships between words based on their semantic features. The participant must select the two words that are the most semantically related from a list of four words (e.g., *run*, *jump*, *read*, *listen*; the correct answers would be “run” and “jump”). This subtest includes 40 items.
- Recalling sentences: This subtest consists of repeating oral sentences of increasing length and complexity. It is comprised of 26 sentences, allowing the assessment of morphosyntactic aptitudes and phonological working memory capacity.
- Formulated sentences: The participant must orally formulate complete and appropriate sentences of increasing complexity using two given words. This subtest, which assesses the capacity to integrate semantic, syntactic, and pragmatic information, consists of 24 items in which an image and a related word are presented to the participant, who must elaborate a sentence that relates both items.
- Semantic relationships: This subtest evaluates the ability to understand sentences by either comparing or identifying related elements. A total of 20 items are presented. In each of them, the participant is asked to choose which two words out of a total of 4 words presented both orally and visually are semantically more suitable to answer to a given question or to complete a sentence (e.g., *One hour is longer than… (a) a minute; (b) a day; (c) a second; (d) a morning*).

The CELF-5 subtests correction was conducted following its standard guidelines. Direct scores for each subtest were transformed into scaled scores to control for the influence of chronological age on participant’s results. The mean of the scaled scores is 10 with a standard deviation of 3, meaning that scores below 7 would be indicative of language difficulties.

#### 2.3.2. Media Use Questionnaire

To collect data on media and media devices use, we applied a questionnaire elaborated ad hoc (see Appendix A). It was composed of six items that were designed to gather information about how many technological devices are used daily by the participants. Moreover, respondents are asked about when, how often, for how long they use them, and the usefulness they perceive in them. This questionnaire also included items on their frequency of linguistic interaction with their parents (see Appendix A).

### 2.4. Procedure

First, we contacted the schools to request their participation in the study. Before starting the assessment, we requested that the parents of all participants included in the sample sign the written consent form.

We first administered individually the four CELF-5 subtests in Spanish, since these were already available and published in this language. After the evaluator ensured that the child had understood the subtest’s instructions, the assessment began with several practice items. After the CELF-5, we administered the self-reported written questionnaire of media use in-group sessions during the school hours.

Analyses were performed using SPSS-25. The relation between media use, the frequency of linguistic interaction with parents, and preteens’ language level was assessed using non-parametric statistics because assumptions for parametric analyses were not met. Independent sample comparisons, Chi-squared tests, and correlations were applied depending on the types of variables considered.

## 3. Results

### 3.1. Descriptive Data

We first conducted independent sample comparisons to ensure that language outcomes were not affected by potential confounding variables, such as sex, family language, or socioeconomic status. No significant differences were found between male and female participants (Word classes: *U* = 384, *p* = 0.759; Formulated sentences: *U* = 324, *p* = 0.486; Recalling sentences: *U* = 382, *p* = 0.784; Semantic relationships: *U* = 324, *p* = 0.489). Family language did not affect language level, and no significant differences were found between participants who had Spanish or Catalan as their family language (Word classes: *U* = 217.5, *p* = 0.456; Formulated sentences: *U* = 230.5, *p* = 0.288; Recalling sentences: *U* = 231.5, *p* = 0.283; Semantic relationships: *U* = 235.5, *p* = 0.242). In addition, no significant differences were found between preteens pertaining to the middle socioeconomic status and those falling into the middle–low class in terms of language level (Word classes: *U* = 398.5, *p* = 0.48; Formulated sentences: *U* = 451.5, *p* = 0.927; Recalling sentences: *U* = 405.5, *p* = 0.542; Semantic relations: *U* = 453.5, *p* = 0.903).

Regarding media use, participants used on average 2.87 technological devices daily, TV being the most frequently used (*n* = 60), followed by smartphones (*n* = 52), computers (*n* = 24), tablets (*n* = 18), PlayStation (*n* = 15), and Nintendo Switch (*n* = 2). See Table 2 for more details about the mean scaled scores and standard deviations of CELF-5 subtests as well as the number of technological devices used.

These devices were mostly used every day, for more than an hour, to watch TV and to play; however, nearly half of the participants did not use any device while eating. Similarly, a little under 50% of the participants stated that they talk much with their parents (see Table 3).

### 3.2. Media Use and Language Level

To explore the potential association between media use and language level, a set of correlation analyses were conducted. Results showed a significant negative correlation between the number of technological devices used daily and language scores for all the four CELF-5 subtests (see Table 4). Furthermore, we found even stronger negative associations between the time of use of these devices and the CELF-5 scores.

**Figure 1:**
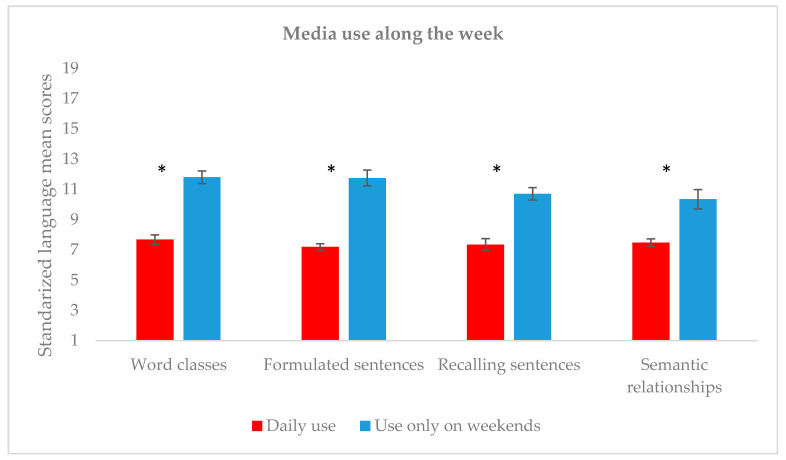
Mean scores on language tasks and use of media along the week. * *p* < 0.001.

Following Figure 1, language scores were significantly lower for preteens who used technological devices every day (*n* = 40) in comparison to those who used them only during the weekends (*n* = 20) (Word classes: *U* = 606.5, *p* < 0.0001, *r* = 0.423; Formulated sentences: *U* = 725, *p* < 0.0001, *r* = 0.674; Recalling sentences: *U* = 672, *p* < 0.0001, *r* = 0.557; Semantic relationships: *U* = 741, *p* < 0.0001, *r* = 0.701). It is also worth noting that participants in the two groups were equivalent in terms of age (*U* = 320, *p* = 0.133).

**Figure 2:**
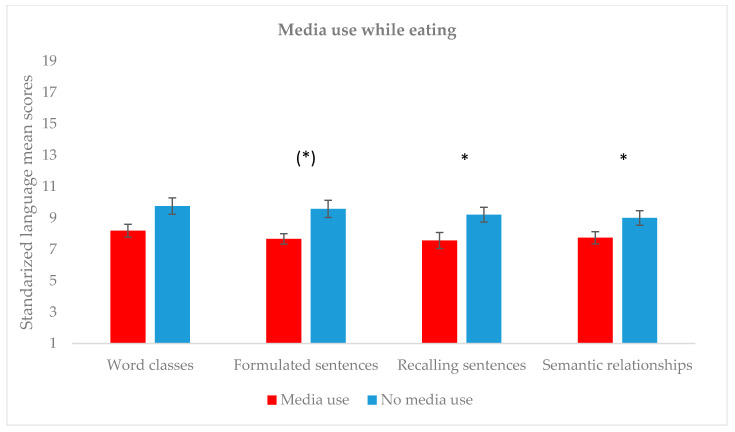
Mean scores on language tasks and use of technological devices while eating. * *p* < 0.05; (*) *p* < 0.06.

Regarding the use of media while eating (see Figure 2), results revealed significantly lower language scores in participants who used media devices during this activity (*n* = 27), as compared to those who did not use them (*n* = 33) in two of the CELF-5 subtests (Recalling sentences: *U* = 584.5, *p* = 0.036, *r* = 0.270; Semantic relationships: *U* = 589.5, *p* = 0.030, *r* = 0.280). Similarly, the subtest Formulated sentences showed a tendency (*U* = 570, *p* = 0.058), and Word classes did not differ between both groups (*U* = 554.5, *p* = 0.101). Again, both groups did not differ with respect to their ages (*U* = 508.5, *p* = 0.262).

**Figure 3:**
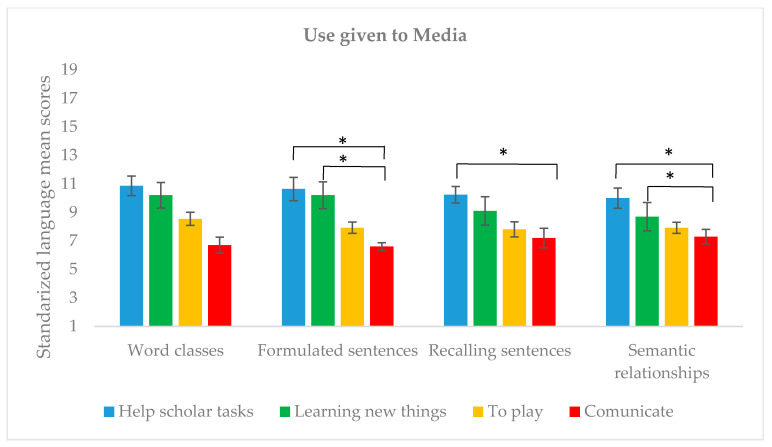
Mean language scores and main use of media. * *p* < 0.01.

To explore the potential differences in language level according to the use given to media devices, independent sample comparisons were conducted with the use given to media as the between-subjects factor (scholar assistance, learning new things, to play, to communicate) on all CELF-5 subtests (see Figure 3). Results yielded significant differences in three of the CELF-5 scores (Formulated sentences: *H* = 13.909, *p* = 0.003, *_ε_*^2^ = 0.235; Recalling sentences: *H* = 13.909, *p* = 0.003, *_ε_*^2^ = 0.15; Semantic relationships: *H* = 13.909, *p* = 0.003, *_ε_*^2^ = 0.268), except for Word classes (*H* = 5.64, *p* = 0.13). Post-hoc analyses evidenced that language scores were significantly higher in preteens who use media for educative purposes. More in detail, post-hoc comparisons revealed that preteens who use media “to help in scholar tasks” or “to learn new things” obtained larger scores in formulated sentences and semantic relationships than those who use it “to communicate” (Formulated sentences: *p* = 0.008, *p* = 0.024, respectively; Semantic relationships: *p* = 0.002, *p* = 0.020, respectively). Similarly, students who use media “to help them with scholar tasks” obtained larger scores in recalling sentences than those who use it “to communicate” (*p* = 0.05). These outcomes were not modulated by participants’ ages, as it did not differ between groups according to the use given to media (*H* = 1.486, *p* = 0.686).

**Figure 4:**
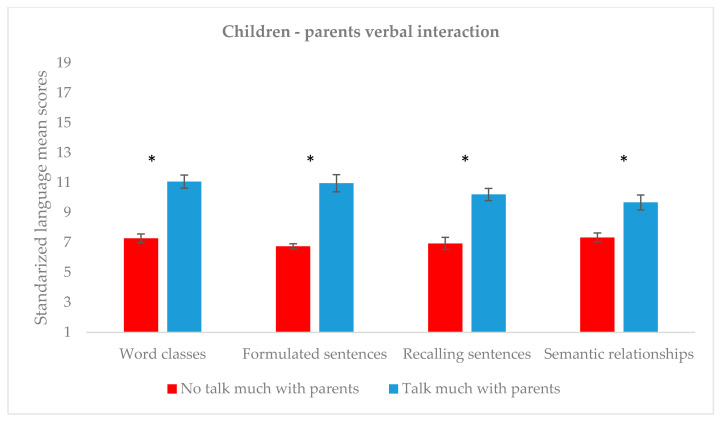
Mean language score and amount of verbal interaction with parents. * *p* < 0.001.

To explore language scores between preteens who stated talking a lot with their parents in comparison to those who reported not talking much with them, independent comparisons were conducted for each language subtest (see Figure 4). More specifically, language scores were significantly higher in participants who talk a lot with their parents in all CELF-5 subtests, as compared to those who do not (Word classes: *U* = 664.5, *p* < 0.001, *r* = 0.419; Formulated sentences: *U* = 840, *p* < 0.0001, *r* = 0.768; Recalling sentences: *U* = 746, *p* < 0.0001, *r* = 0.577; and Semantic relationships: *U* = 808.5, *p* < 0.0001, *r* = 0.699). These results were not affected by age, as participants in both groups did not differ in this regard (*U* = 396, *p* = 0.356).

**Figure 5:**
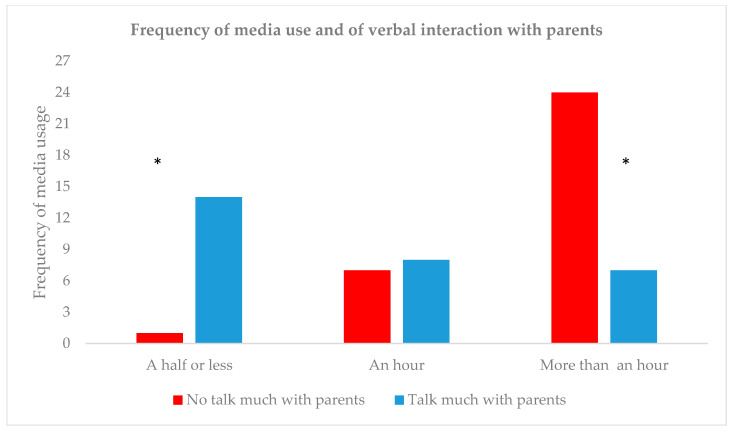
Frequency of media use in hours in relation to the amount of interaction with parents. * *p* < 0.0001.

Finally, the frequency distribution analysis between the use of media in minutes and the tendency to talk with the parents (see Figure 5) showed that participants who considered that they did not talk much with their parents used technological devices in higher rates of time (*χ²* (2,60) = 19.495, *p* < 0.0001). Post-hoc tests using standardized residuals revealed that among preteens with the lowest amount of daily use of media, there were significantly more participants who talk frequently with their parents (Z_adj_ = 3.96, *p* < 0.001). In addition, participants who spent the highest rates of time with media every day report talking with their parents less frequently (Z_adj_ = 3.87, *p* < 0.001). There were no differences in terms of the frequency of self-reported interaction with their parents among preteens who spend intermediate time levels with media per day (one hour per day) (Z_adj_ = 0.6, *p* = 0.835).

## 4. Discussion

This study aimed to evaluate the associations between media use and language skills in a sample of preteens aged 11 and 12. As a secondary objective, this study assessed the potential impact of the quantity of parental language input on preteens’ actual language skills.

Regarding technological use, most participants in our sample make daily use of technological devices. Participants stated that they used between 2 and 3 devices daily, on average. In particular, all of them had a TV at home, which was the most used device, and the second most used was the smartphone. Considering that almost 90% of the participants have a smartphone, we can consider that this sample has media as a daily element of their lives. Regarding this massive use of technological devices, recent studies have shown that the smartphone is the most used device among the youth between 10 and 14 years of age [4], which is a result that is not completely mirrored by the results of the present study, given that TV was the most frequently used one. Participants in the present study stated that they use technological devices for more than an hour and, mostly, to play. Regarding whether or not they use the devices while eating at these ages, approximately half of the participants in our sample did use them.

In terms of language skills, scores on the language subtests showed that values are slightly inferior to those expected when standardizing, which should commonly yield a mean score of 10 and a standard deviation of 3 points. We speculate that this might be because we only included bilingual participants with middle or low socioeconomic status.

We also report a negative association between the number of devices, their time of usage, and the CELF-5 scores. Considering the time of usage, results in all language subtests were lower for participants who made daily use of technological devices, as compared to those who made a more restricted use, mostly on weekends. These results are in consonance, but nuanced, with recent studies showing that children who use a moderate amount of media show the largest language gains, whereas both the lowest and the highest levels of media use are associated with smaller language gains [30]. In addition, other studies have demonstrated that sustained exposure to screens at an early age could even increase the risk of language delay [36]. Nevertheless, it has also been shown that when parents or caregivers are involved in the use of technological devices (frequent media joint engagement), the negative relationship between media exposure and language development has not been found [10].

Another important aspect of this study concerns the self-reported use of technological devices during meals. Recent studies have shown that children who regularly use technological devices during mealtime show a delay in language development, as long as this media use may displace language-enhancing activities, such as the interaction with their parents and siblings [30]. In our study, and considering that all participants are preteens, this interaction still shows an effect, as participants who eat while using technological devices show lower results in language subtest than those who do not combine these two activities. In this vein, several experts recommend limiting the use of technologies due to its negative effect on child development, not only in terms of language and cognition but also on their daily habits and routines, such as eating or sleeping [19,36].

In terms of the different uses given to media, results showed that those participants who stated to use media mainly to assist with their homework or to learn new things had higher scores on most of the language subtests as compared to the participants who stated to use media mainly to communicate with other people. In this vein, a meta-analysis examining the effects of children’s exposure to international co-productions of Sesame Street, a program with clear learning content, showed significant positive effects of exposure to the program in cognitive outcomes [37]. Other studies have also shown that media used with educational purposes can improve language development, especially in economically disadvantaged children [11]. Therefore, the use given to media can influence language skills, which might mitigate their negative effect or even boost language development.

Another factor seems to mediate the relationship between media and language development. In this study, participants were also asked about their perceptions of the amount of interaction with their parents. Approximately half of the sample did not perceive to have much linguistic interaction with their parents or caregivers, and results showed that their language scores were lower as compared to those who reported having frequent interaction with them. More in detail, participants who reported having less communication with their parents spent more time using electronic devices. Thus, the interaction between parental language input and children’s language level arises as a fundamental factor for the adequate development of language. Previous studies support the view that both the interaction with and the exposure to media are decisive factors for language development and suggest that they are directly related [10]. There is also evidence showing that when the interaction between parents and children is conditioned by the simple presence of a technological device in the background, the quantity and quality of the language used seems to be diminished [24].

We suggest that the preadolescent population might be making an abusive use of media and, adding the use given to it together with a low interaction with their parents or caregivers, can lead to an impoverishment in their language skills. Hence, we can conclude that the linguistic skills of preteens who have been considerably exposed to technological devices exhibit deficiencies and are below the average level. Nevertheless, we cannot claim that this relationship is causal, since other studies have described a larger motivation to use media for communicative purposes in children with language difficulties. In this regard, adolescents with language difficulties use media to establish social relationships and communicate, given that these communication formats (instant messaging and e-mails) are more impersonal and tolerant with linguistic errors [38]. However, this same study did not find differences in the frequency of media usage between children with language problems and their normative peers. Instead, other studies suggest that parents of children with language difficulties tend to talk less with them [39] and produce a language of worse quality [40]. Therefore, it seems that the relation between the use of media, the interaction with parents, and adolescent’s language level is rather complex. A bidirectional cyclic relation might work as a more suitable explanation, in which the different factors influence each other. In this sense, we speculate that the use of media would diminish the time of parent–adolescent interaction and would worsen its quality. In a complementary way, having language difficulties might generate a weaker tendency by others to carry out appropriate communicative behavior. In turn, this behavior would lead to increased use of media by adolescents with language difficulties in an attempt to palliate this effect.

In general terms, the present results confirm the generalized spread of the use of media and, thus, the need to study its effect on children and adolescents, especially in those with prolonged exposures. Nevertheless, to the extent that parents or caregivers use technological devices with their children to learn, the negative effects of media on language development can be reduced, as the joint participation in learning activities with these devices can act a mitigating factor for the potential risks of exposure to media [30,41].

However, the results of this study should be taken with caution due to several limitations. The first limitation is the small sample size. Second, the Questionnaire on the Usage of Media in the Family Setting has some questions that offer little variability in their response items. In particular, for questions regarding the time spent daily using media—with the higher score (more than one hour) including around 50% of participants—and the question regarding frequency of talking with parents—with a dichotomous answer. Third, only parent’s language input has been analyzed in this study not considering other agents such as siblings or peers. Finally, data collection was merely based on self-perceptions on the use given to media, as well as the linguistic input that preteens consider receiving from their parents. Consequently, the data used in this study are based on the associations between self-reports and objective linguistic outcomes.

Hence, future investigations might benefit from evaluating the quality and quantity of language input from parents, siblings, and peers through an observational approach, being advisable to include objective measures on the time spent using media devices to explore its potential associations with the language development of preteens in a larger sample. A more detailed differentiation regarding the duration of use of media devices and the frequency of talking with parents and other agents is needed in future research.

## 5. Conclusions

To summarize, the data obtained in this study point to a generalized use of media devices among preteens of 11 and 12 years of age, which is related to lower language scores and a perception of less frequent communication with their parents. Therefore, it can be considered that media has an influence on language skills depending on both the time spent using it and the type of use given to it. However, it must be kept in mind that media use and language level do not have a direct causal relation but rather a cyclic relation in which the involved factors mutually affect each other.

Considering our results, we recommend limiting the use of media devices to less than an hour per day and with an academic purpose, and to use them for communicative and play only on weekends.

## Figures and Tables

**Table 1:** Sociodemographic data of the participants.

	Frequency
**Age**	
11	22
12	38
**Sex**	
Female	17
Male	43
**Socioeconomic status**	
Low	27
Middle	33
High	0
**Family predominant language**	
Spanish	53
Catalan	7

**Table 2:** Mean scaled scores and standard deviations for language subtests and the number of devices used by the complete sample (*n* = 60).

	Mean	SD
**CELF-5**		
Word classes	9.05	2.740
Formulated sentences	8.72	2.762
Recalling Sentence	8.47	2.771
Semantic relationships	8.43	2.459
**Number of devices used**	2.87	1.186

Note: SD = standard deviation.

**Table 3:** Frequency and percentage of media use and communication with parents.

	Frequency	Percentage
**Media use**		
Daily	40	66.6%
Only weekends	20	33.3%
**Usage time**		
Less than half an hour	3	5%
Half an hour	11	18.3%
One hour	15	25%
More than one hour	31	51.6%
**Use given to media devices**		
Assistance in school tasks	14	23.3%
Learning new things	10	16.6%
Playing games	26	43.3%
Communication	10	16.6%
**Devices used while eating**		
None	33	55%
Television	19	31.6%
Other devices	8	13.3%
**Talking a lot with their parents**		
No	32	53.3%
Yes	28	46.6%

**Table 4:** Spearman correlations between the number of devices, time of media use, and language scores.

	1	2	3	4	5	6
1. Number of devices	1					
2. Time of media use ^a^	0.507 **	1				
3. Word classes	−0.273 *	−0.347 *	1			
4. Formulated sentences	−0.395 **	−0.714 **	0.499 **	1		
5. Recalling sentences	−0.358 **	−0.504 **	0.844 **	0.732 **	1	
6. Semantic relationships	−0.395 **	−0.776 **	0.523 **	0.945 **	0.76 **	1

^a^ Time of use per day; * *p* < 0.05; ** *p* < 0.01.

## Appendix A. English Translate Version of the Questionnaire on the Usage of Media in the Family Setting

1. Which technological devices do you use every day (you can choose more than one option)?
  - Tablet
  - Computer
  - Television
  - Mobile phone
  - Nintendo Switch
  - PlayStation
  - Others: _________________________
2. How many hours do you think you approximately spend using these devices daily?
  - Less than half an hour
  - Half an hour
  - One hour
  - More than one hour
3. You use media devices…
  - Everyday
  - Weekends or festivities only
4. Do you usually use these devices while eating?
  - Yes, the television
  - Yes, other technological devices
  - No, I don’t
5. Do you consider that you talk a lot with your parents?
  - Yes, I do
  - No, I don’t
6. Which is the most frequent usage you give to these devices? (Choose only one option)
  - Assistance in school tasks
  - To learn new things
  - To play
  - To communicate with friends or relatives

Thanks for your participation!
